# Process evaluation of a social franchising model to improve maternal health: evidence from a multi-methods study in Uttar Pradesh, India

**DOI:** 10.1186/s13012-018-0813-y

**Published:** 2018-09-24

**Authors:** Loveday Penn-Kekana, Timothy Powell-Jackson, Manon Haemmerli, Varun Dutt, Isabelle L. Lange, Aniva Mahapatra, Gaurav Sharma, Kultar Singh, Sunita Singh, Vasudha Shukla, Catherine Goodman

**Affiliations:** 1 London School of Hygiene and Tropical Medicine, 15-17 Tavistock Place, London, WC1H 9SH UK; 2 Sambodhi Research and Communications, Noida, Uttar Pradesh India; 3 Independent Consultant, Delhi, India

**Keywords:** Process evaluation, Multi-methods, Social franchising, Maternal health, India

## Abstract

**Background:**

A prominent strategy to engage private sector health providers in low- and middle-income countries is clinical social franchising, an organisational model that applies the principles of commercial franchising for socially beneficial goals. The Matrika programme, a multi-faceted social franchise model to improve maternal health, was implemented in three districts of Uttar Pradesh, India, between 2013 and 2016. Previous research indicates that the intervention was not effective in improving the quality and coverage of maternal health services at the population level. This paper reports findings from an independent external process evaluation, conducted alongside the impact evaluation, with the aim of explaining the impact findings. It focuses on the main component of the programme, the “Sky” social franchise.

**Methods:**

We first developed a theory of change, mapping the key mechanisms through which the programme was hypothesised to have impact. We then undertook a multi-methods study, drawing on both quantitative and qualitative primary data from a wide range of sources to assess the extent of implementation and to understand mechanisms of impact and the role of contextual factors. We analysed the quantitative data descriptively to generate indicators of implementation. We undertook a thematic analysis of the qualitative data before holding reflective meetings to triangulate across data sources, synthesise evidence, and identify the main findings. Finally, we used the framework provided by the theory of change to organise and interpret our findings.

**Results:**

We report six key findings. First, despite the franchisor achieving its recruitment targets, the competitive nature of the market for antenatal care meant social franchise providers achieved very low market share. Second, all Sky health providers were branded but community awareness of the franchise remained low. Third, using lower-level providers and community health volunteers to encourage women to attend franchised antenatal care services was ineffective. Fourth, referral linkages were not sufficiently strong between antenatal care providers in the franchise network and delivery care providers. Fifth, Sky health providers had better knowledge and self-reported practice than comparable health providers, but overall, the evidence pointed to poor quality of care across the board. Finally, telemedicine was perceived by clients as an attractive feature, but problems in the implementation of the technology meant its effect on quality of antenatal care was likely limited.

**Conclusions:**

These findings point towards the importance of designing programmes based on a strong theory of change, understanding market conditions and what patients value, and rigorously testing new technologies. The design of future social franchising programmes should take account of the challenges documented in this and other evaluations.

## Background

### Introduction

India’s private health sector is extensive and diverse. It ranges from sophisticated tertiary hospitals providing medical care of an international standard to alternative systems of medicine and unqualified rural health providers. The majority of registered doctors work in the private sector, which is often the first point of contact for a substantial proportion of the population [1–3]. Regulating the private sector in India has proved challenging, and alternative strategies that encourage private providers to raise standards are required [4]. However, there remains limited evidence on the most effective strategies to improve the quality of private sector services [5–8].

One proposed strategy is social franchising, an organisational model that applies some of the principles of commercial franchising to support the provision of branded, quality-assured services of social importance, such as healthcare, via a network of private providers [6]. Although there is considerable variation across programmes, Viswanathan et al. [9] identify a set of core characteristics that most have in common: (1) a third party administrator, typically an NGO which manages the brand and supervises the network providers through regular visits and audits; (2) the use of protocols and guidelines under which providers must operate; (3) a focus on the sale of healthcare services, in addition to healthcare commodities; (4) the aim of providing quality-assured health services to the most under-served populations; and (5) the aim of achieving self-sustainability both from the franchisor and franchisees’ perspectives. The social franchising model has been applied to a wide range of health-related services and is one of the fastest growing private sector interventions in recent years, with 83 active healthcare social franchising programmes identified in 2015 in low- and middle-income countries (LMICs), 37 of which began between 2007 and 2012 [9].

Although considerable resources are being channelled into social franchising in LMICs, there is limited rigorous evidence on the effectiveness of clinical social franchising. Moreover, while impact evaluations are needed to measure the effect of complex interventions, process evaluations also have an important role to play in examining “the context and implementation of an intervention, the mechanisms through which it may affect outcomes, and the response of the intervention target population” [10]. When conducted alongside impact evaluations, process evaluations can help explain the “black box” behind complex and multi-faceted interventions, leading to an understanding of the mechanisms underlying their impact (or lack of impact) and thus to the provision of more relevant policy recommendations.

The focus of this paper is the Matrika social franchising model in Uttar Pradesh, India. The programme aimed to improve quality and coverage of maternal health services in three districts of the state. The main element of the programme was the “Sky” social franchise. In previous research, we examined the population impact of the programme using a rigorous quasi-experimental design [11]. Findings from the impact study show that the multi-faceted programme was not effective in improving the quality and coverage of maternal health services [12]. It had no significant effect on facility births, the primary outcome. Across an additional 56 pre-specified outcomes, measuring healthcare use, content of care, patient experience, patient knowledge, healthy behaviours, and financial strain, there was no difference between intervention and comparison areas. There was some evidence of an increase in recommended delivery care practices.

This paper reports findings from a process evaluation, conducted alongside the impact evaluation, with the aim of explaining why the Matrika social franchise programme did not have a population impact. Using a theory of change and multiple sources of data, we examine implementation of the programme, the mechanisms of impact and contextual factors to understand why the programme did not improve the quality and coverage of maternal health services. Although the Matrika programme was multi-faceted, we focus on its core component, the Sky social franchise network.

### Social franchise model

The Matrika programme was a complex multi-faceted intervention which sought to improve maternal health primarily by reducing deaths from post-partum haemorrhage. The programme combined various activities to encourage more women to use services and raise the quality of antenatal care (ANC), obstetric care, and family planning services. The intervention was implemented by World Health Partners (the franchisor) in partnership with Pathfinder International. It officially started in March 2013 and ended in May 2016, with a budget of USD 3,250,000.

The rationale for the programme was primarily based on a pragmatic argument that private health providers, since they are widely accessible and frequently used, should be harnessed to improve the health of women during pregnancy and childbirth. However, it was also recognised that their capacity and links with the public sector required strengthening and that there was also inadequate demand for maternal health services. Hence, there was a need for a multi-pronged approach to address both supply- and demand-side constraints to coverage of quality maternal health services.

**Fig. 1:**
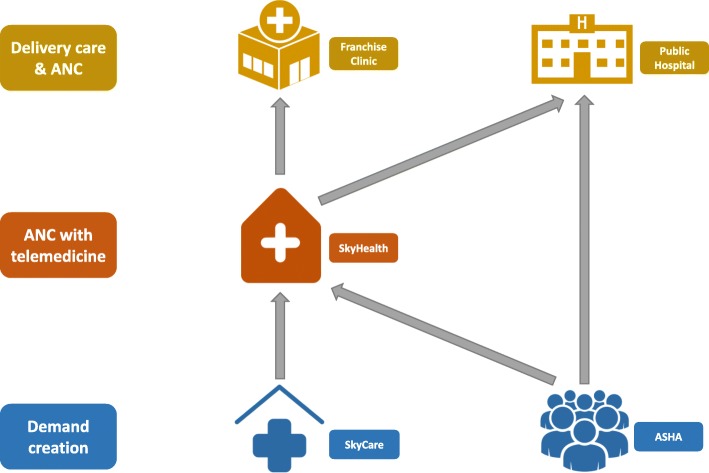
Social franchise network of providers and patient flow

The franchisor engaged with private for-profit health providers by establishing the Sky social franchise network. Recruitment of providers into the social franchise involved visiting prospective providers to establish their interest, explaining the benefits of joining, and signing a contract to formalise the relationship. The network comprised private providers at three levels: SkyCare providers, SkyHealth centres, and Franchise Clinics (Fig. 1). SkyCare providers made up the lowest level of the network. They were informal rural health providers, many of whom were medically unqualified working out of their home. Their role within the franchise was primarily to encourage women to use services at higher levels in the network, and they received financial incentives where this was successful for ANC. The franchisor also trained SkyCare providers to conduct mobile phone consultations with a central medical facility in Delhi, where they employed qualified doctors to conduct remote medical consultations. In the initial phase of implementation, the franchisor recruited government community health workers, known as accredited social health activists (ASHAs), to be SkyCare providers, but this was stopped due to objections from the government.

The second level was SkyHealth centres. These were small clinics owned by individuals who mostly had a qualification in ayurveda, yoga and naturopathy, unani, siddha, or homoeopathy (AYUSH). Their main role within the franchise was to provide ANC consultation, for which they received a financial incentive from the franchisor, and to channel clients towards appropriate facilities for delivery. ANC consultations were to be free to women, though SkyHealth centres could charge for any commodities provided. Most SkyHealth centres were new to ANC provision. During the design stage, there was an expectation that these providers would also provide delivery care within the franchise, but this was vetoed because of concerns about their skill levels. SkyHealth centres were expected to conduct their ANC consultations using telemedicine over the Internet with doctors in the central medical facility, for which they were required to purchase a computer and other equipment. A “medical diagnostic box” allowed them to measure and communicate clinical information, such as blood pressure and pulse, to the central medical facility. SkyHealth centres were also integrated into the public supply chain of iron and folic acid for provision during ANC. At the highest level, Franchise Clinics^1^ were private hospitals offering delivery and emergency obstetric care under a fee structure set by the franchisor. The franchisor gave branded signage to providers at all levels and marketed the Sky brand through various channels such as wall paintings and radio spots.

To improve and standardise quality of care, clinical training of two to three days and regular quality improvement (mentoring) visits were given to SkyHealth centres and Franchise Clinics (not SkyCare). Although not part of the social franchise network, public facilities in the same districts were identified to act as referral destinations for delivery care and were also given training. SkyHealth centres were trained to provide ANC, recognise and stabilise pregnancy complications, facilitate timely referrals, and provide post-partum contraception counselling. They were also trained in how to operate the telemedicine equipment. Training of Franchise Clinics and public providers covered the same topics as well as emergency obstetric care.

To increase the demand for maternal health services, there were village-level information activities such as wall paintings, billboards, radio spots, and film shows. There was also a 1-day training of ASHAs on birth preparedness, recognition of danger signs during pregnancy, and appropriate sources of ANC and delivery care, with the intention that ASHAs would encourage women to use both public facilities and facilities within the Sky network.

By the end of the programme, 365 SkyCare providers, 50 SkyHealth centres, and 8 Franchise Clinics were part of the network. Clinical training lasting 2 to 3 days was given to 58 private providers, and there were 225 quality improvement visits in 50 private facilities. One day of training was given to 2149 ASHAs.

## Methods

### Study setting

Uttar Pradesh is the most populous state in India with almost 200 million people in 2011, now estimated to be over 220 million. The population is predominantly rural (77%). Maternal mortality remains high in Uttar Pradesh, with the most recent estimate at 285 deaths per 100,000 live births [13]. The proportion of women giving birth in a health facility has risen rapidly in the past decade, from 39% in 2005–2006 to 79% in 2015–2016 [14]. The public sector accounted for much of the increase, rising from 7 to 45% over the same period. Women with four or more ANC visits increased from 37% in 2005–2006 to 51% in 2015–2016.

The Sky social franchise was implemented in three adjoining districts (Kanpur Nagar, Kanpur Dehat, and Kannauj) in Uttar Pradesh. With Kanpur as its largest city, Kanpur Nagar district is the most populated of the three, with 4.6 million people. With 1.7 and 1.8 million inhabitants, respectively, Kannauj and Kanpur Dehat districts are predominately rural. The private market for maternal healthcare in the study areas is largely made up of small, individually owned hospitals and clinics located in urban and peri-urban areas [15]. Most facilities are owned by doctors and, to a lesser extent, AYUSH providers.

### Evaluation framework

Our approach was guided by the Medical Research Council process evaluation framework, which argues that “an understanding of the causal assumptions underpinning the intervention and use of evaluation to understand how interventions work in practice are vital in building an evidence base that informs policy and practice” [16]. The framework gives prominence to three components of a process evaluation: (1) implementation, (2) mechanisms of impact, and (3) context.

**Fig. 2:**
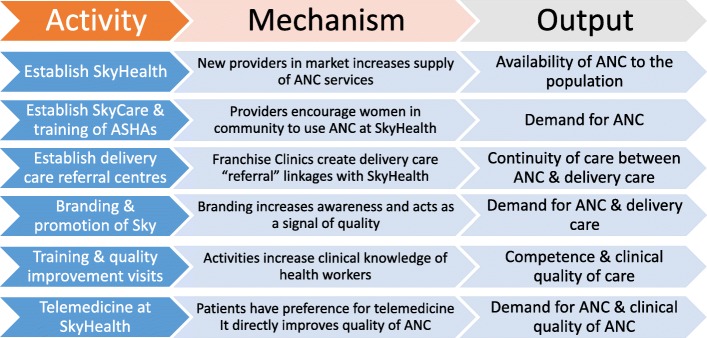
Theory of change showing the mechanisms linking inputs and outputs

Our independent, external evaluation began with an initial meeting between the evaluation team and the implementing partners in December 2013. Over the course of implementation, we updated our knowledge on activities undertaken and developed hypotheses about the potential pathways of impact. For the purposes of this paper, we highlight six key mechanisms through which the programme was hypothesised to link activities to outputs, as summarised in Fig. 2. These outputs—by improving the demand, supply, and continuity of maternal health services—were anticipated to improve a range of outcomes along the continuum of care, including measures of healthcare utilisation, quality of care (content of care, health provider practices, patient experience), patient knowledge, healthy behaviours, and financial strain [12]. Taken together, these outcomes were designed to capture both the potential benefits and the unintended consequences of the programme. While this theory of change is inevitably a simplification of reality, particularly in terms of the nature of the relationship between inputs and outputs, it provides a useful starting point for understanding how the programme was intended to work.

The theory of change provided our analytical framework. Specifically, we analysed the data and organised the results according to the six key mechanisms in Fig. 2, as a means of explaining the success or failure of the programme in generating the desired outputs, ultimately with a view to understanding why the social franchise had no measurable effect on population outcomes. We explored the potential reasons for the impact evaluation results by comparing our findings with the theory of change, paying particular attention to the influence of implementation, mechanisms, and context. The success of the Sky social franchise depended on these mechanisms operating in practice, which in turn required a number of assumptions to hold. Some of these assumptions were identified a priori, while others were explored inductively.

### Data

We undertook a multi-methods study [17], drawing on both quantitative and qualitative primary data. We relied primarily on the quantitative data to assess the extent to which components of the intervention were implemented. Both qualitative and quantitative data were used to understand mechanisms and the role of contextual factors. While these two types of data collection were done by different groups of researchers, the development of the study protocol and the design of tools involved input from all the researchers [11].

There were five distinct data collection activities (Table 1). These included: (1) two rounds of a household survey with women who had given birth in the previous 2 years, covering villages in intervention areas (with a SkyCare or SkyHealth facility) and comparison areas. The interview covered questions about the source of ANC, delivery care and family planning, referral by ASHAs, and knowledge of the Sky brand; (2) two rounds of a survey with health providers, covering information about the facility (branding, use of telemedicine), frequency of training and monitoring visits, and ANC knowledge; (3) clinical observations of ANC telemedicine consultations in SkyHealth centres and Franchise Clinics based on WHO’s minimum care package of interventions during pregnancy [18, 19]; (4) survey of social franchise users selected from SkyHealth centres and Franchise Clinics covering questions on socio-economic characteristics, women’s pathway of care, experience with telemedicine, and costs and perceptions of services; and (5) participant observations and semi-structured interviews with social franchise staff, franchised providers, and women who had used social franchise facilities to provide an in-depth understanding of the functioning of the model and perceptions of its main actors and beneficiaries.

**Table 1:** Description of data collection methods

Tools and date of data collection	Sampling strategy	Target population	Information captured
Household surveys (two rounds—Jan 2015 and May 2016)	3600 (round 1) and 3452 (round 2) households in 180 study clusters. Three types of cluster were surveyed. Group A contained clusters with a SkyCare or SkyHealth provider in the three intervention districts. Group B comprised clusters with no social franchisee in the same three districts. Group C was taken from neighbouring districts that did not have any social franchise network operating within them	Eligible respondents included all women aged 15–49 years who gave birth in the previous 24 months (round 1) or 18 months (round 2), including those who had a stillbirth or whose child died since birth. Eligible women were identified through a census of households, conducted 1 month before the household survey	- Source of care received for ANC, delivery care, and family planning - Whether the woman was accompanied by an ASHA for ANC and delivery care - Community awareness of the Sky brand
Health provider surveys (two rounds—Jan 2015 and May 2016)	Using a census of all health providers within the study clusters, we randomly selected for interview one private health provider (social franchisee in intervention clusters), one government health provider, and one ASHA in each cluster. In the second round, we sought to re-interview the same providers or, if not available, a random replacement of the same type. Complete interviews were obtained from 454 health providers in round 1 and 446 interviews in round 2	For the purposes of the census, we defined a health provider as any institution or individual whose primary purpose is to provide healthcare. We excluded drug sellers	- Branding of the facility - Use of the telemedicine and mobile phone consultations - Training, supervision, and monitoring - Health provider knowledge of ANC and actual practice of ANC based on respondents’ recall of their most recent ANC consultation
Clinical observations of antenatal care consultations (Feb–Aug 2016)	A purposive sample of six facilities (4 SkyHealth and 2 Franchise Clinics) was selected to reflect variation amongst facilities within the network. This was not intended to be a representative sample that would allow generalisation across the whole network	25 observations of ANC visits using telemedicine	Clinical quality of care as defined by the minimum care package of interventions required during pregnancy recommended by WHO [18, 19]
Social franchise user survey (Apr–Jun 2016)	15 health facilities were selected based on stratified random sampling (9 SkyHealth facilities, 6 Franchise Clinics). 760 women were selected from facility records	Eligible women were those who had received ANC or delivery care from a social franchise facility within the previous year and, in the case of ANC, had given birth by the time of the survey	- Socio-economic characteristics of women and their household - Pathway of care - Experience with telemedicine - Perception of services and costs
Qualitative research: semi-structured interviews and participant observations (including conversations and other interactions) (2016)	From the 15 focal sites that were randomly sampled for the social franchise user survey, we purposively selected 6 “intensive” sites to reflect differences in number of clients and types of services offered: 2 Franchise Clinics and 4 SkyHealth centres We used a combination of clinic lists and the help of ASHAs to identify women for interview. We purposefully sampled women to reflect a representation of different profiles based on age, parity, level of education, and urban, peri-urban or rural residence	Participant observations and semi-structured interviews with 30 women; 21 ASHAs; 15 SkyCare providers; 11 SkyHealth directors who were still part of the Sky franchise and 5 who had either not joined, left, or been asked to leave; 3 central medical facility staff and 9 franchisor staff	- Topics with franchisees included motivation for joining social franchise, how being part of the social franchise impacted their business and the care they provide, how sustainable the social franchise model is, differences between private and public sectors, and perspectives on their integration - ASHAs: understanding of social franchise model, traditional responsibilities of an ASHA and how tasks with the social franchise fit into their role, incentives and payment systems, practices of working with public and private sectors - Women: care-seeking behaviours and decision making in pregnancy and childbirth, perceptions of the interactions with social franchise providers (including telemedicine), ASHAs, and the public sector - Programme staff: conceptualisation of the model, challenges and successes during implementation, modifications made, financial and sustainability considerations of the social franchise, nature of interactions with stakeholders and partners

Survey tools were translated from English into Hindi and back-translated, before being piloted in the field. The semi-structured interviews were carried out using topic guides that were pilot tested and adapted in the course of data collection. These adaptations were based on regular discussions of emerging themes between the four members of the qualitative research team and in response to findings from the quantitative data. Interviews were transcribed and translated into English using a professional translation service and checked by one of the native Hindi-speaking members of the research team.

### Analysis

We analysed the quantitative data descriptively to generate indicators of implementation and provide insights as to the context surrounding the intervention, reporting means and proportions, as appropriate. Indicators of implementation were grouped according to social franchise recruitment and branding, community-level measures, and provider-level measures of implementation. Individual indicators of provider antenatal care knowledge and practice were combined to generate summary indices, for which we report the mean for different types of health provider in intervention and comparison areas. All quantitative data were analysed in Stata 14.2.

All qualitative interview transcripts were coded using NVivo 10.0 software according to a priori themes related to our evaluation research questions. Further themes were added throughout analysis after familiarisation with the context and data, and coding was stratified by facility and respondent. A simultaneous evaluation of another social franchise in Rajasthan with similar methods gave our findings a point of comparison, which we used to deepen our understanding of the results in this study. As such, analysis was a continuous process. After undertaking a preliminary thematic analysis of the interviews and field notes [20], we held a number of reflective meetings including researchers involved in all components of the process evaluation to triangulate across data sources, synthesise evidence, and identify the main findings [17].

We validated preliminary findings through discussion with the implementers of the intervention. We produced a report which we sent to implementers for comment and discussion before finalising. We fed back more finalised results to policymakers and other key stakeholders in India. Insights generated from these interactions were used to finalise the results reported in this paper.

## Results

We highlight key findings under each of the six broad mechanisms identified in the theory of change. In each section, we start by outlining the potential mechanisms of impact involved. We then explain the main findings, bringing together evidence on the degree of implementation, the logic of potential mechanisms of impact, and the influence of the context in which the intervention was implemented.

### Competitive nature of the market for ANC meant franchise providers achieved very low market share

Establishing SkyHealth facilities within the social franchise network was designed to increase the availability of ANC in rural areas and to provide a point of contact for women to be referred for delivery care in both the public and private sectors.

**Fig. 3:**
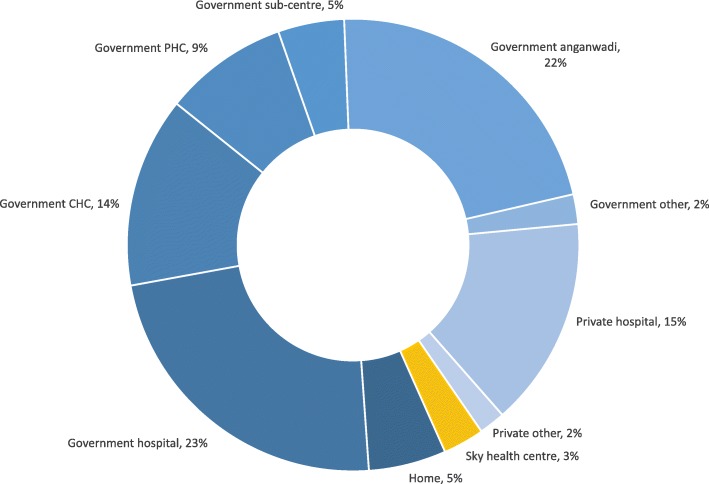
Share of market by source of care (type of facility) for antenatal care. Notes: Source of data is the household survey of women. Data are from intervention clusters only after the start of social franchise. Anganwadi centres are government providers offering basic ANC at the village level. They constitute the lowest level of public health system. PHC primary health centre, CHC community health centre

Social franchise providers achieved very low market share in areas in which they operated. Data from the household survey show that SkyHealth centres captured only 3% of the market for first ANC visits amongst women living in close proximity (Fig. 3). There are a number of possible reasons for the low market share but the most likely relates to context, with the market being much more competitive than anticipated. The fact that 75% of women sought ANC from the public sector suggests that women were willing to travel to seek public care even when public facilities were not available in their village (Fig. 3). In terms of private sector competition, the census of health providers indicated that SkyCare and SkyHealth providers combined represented just 13% of all private providers in their area (Table 2).

**Table 2:** Social franchise recruitment and branding

Indicator	Provider census/survey (January 2015)
Sky providers as proportion of private providers in intervention clusters (%)	49/382 (13%)
Sky providers as proportion of all providers in intervention clusters (%)	49/515 (10%)
Sky providers who would recommend others to join social franchise (%)	28/49 (57%)
Sky providers who are male (%)	43/49 (88%)
Sky provider branded (%)	49/49 (100%)

Notes: Sources of data are the census of health providers (January 2015) and the health provider survey (round 1, January 2015). Data are *n*/*N* (%)

Consistent with the competitive environment and multiple care-seeking options, women’s pathway of care was found to be fragmented in the sense that they often used more than one provider for ANC. Data from the social franchise user survey showed that women who visited SkyHealth centres made on average 4.7 visits for ANC, and out of these, only 1.5 were visits to SkyHealth centres. The data show that these women relied more on the public sector for ANC, particularly healthcare provided at Anganwadi centres, which distributed iron and folic acid as well as tetanus toxoid vaccination at the village level for free. The fragmented care seeking was exacerbated by ASHAs being incentivised to bring women to various types of facilities and sectors. SkyHealth providers often paid ASHAs to bring clients to them, as is standard practice in the rest of the private sector [15], something that was informally acknowledged by the Sky franchisors who labelled it as reimbursement for ASHA transport costs. Qualitative research revealed that ASHAs would sometimes organise ANC trips for women involving visits to both government and SkyHealth facilities on the same day. Women interviewed in the qualitative research and those observed during the fieldwork often came to SkyHealth providers with ASHAs directly from the public sector.

The low market share suggests that the price subsidies for ANC provided in SkyHealth centres were not sufficient to attract women in reasonable numbers to their facilities. We also note that the vast majority of Sky providers were male when women are known to have a preference for female providers (Table 2).

### Awareness of the franchise was low and the brand was not seen as a signal of quality

Branding and other promotional activities sought to raise demand for ANC and delivery care offered by facilities in the social franchise network. The mechanism depended critically on patients knowing about the brand and recognising it as a signal of quality.

Of the SkyCare and SkyHealth facilities surveyed, all were branded (Table 2). However, household survey data indicate that the Sky brand was not recognised by most women in the community (Table 3). In villages with a Sky provider, less than a quarter of women recognised the franchise logos suggesting that the vast majority of women were not exposed to the potential influence of the branding on healthcare-seeking behaviour (Table 3). Moreover, only one in ten women reported knowing that there was a Sky provider in their village (Table 3).

**Table 3:** Community-level measures of implementation

Indicator	Household survey (May 2016)
A. Awareness of social franchise amongst women in areas with franchise provider	
Heard of Sky brand (%)	212/1163 (18%)
Recognise SkyCare logo (%)	282/1165 (24%)
Recognise SkyHealth logo (%)	273/1165 (23%)
Reported knowing a provider in village who was part of franchise (%)	132/1165 (11%)
B. Awareness of social franchise amongst women in districts without franchise	
Heard of Sky brand (%)	30/1140 (3%)
Recognise SkyCare logo (%)	41/1143 (4%)
Recognise SkyHealth logo (%)	48/1143 (4%)
Reported knowing a provider in village who was part of franchise (%)	5/1143 (0%)
C. Maternal health seeking accompanied by ASHAs	
Women using ANC accompanied by ASHAs in intervention districts (%)	1386/1977 (70%)
Women using ANC accompanied by ASHAs in comparison districts (%)	686/1018 (67%)
Women giving birth in facility accompanied by ASHAs in intervention 2003districts (%)	1165/1715 (68%)
Women giving birth in facility accompanied by ASHAs in comparison districts (%)	585/892 (66%)

Notes: Source of data is the household survey of women (round 2, May 2016). Data are *n*/*N* (%)

These data were supported by the participant observations at facilities which demonstrated that the prominence of Sky branding varied greatly. A common complaint from providers was that the franchisor had not delivered on their promise to market the brand. What marketing had been done was said to be insufficient and often to have been poorly implemented. Some talked about how they had to take the initiative into their own hands and produce their own leaflets and organise health camps in villages (health promotion activities and check-ups in villages).

I am working on a zero balance here also. If we do not do anything of our own, on the side, then we would, I mean WHP can make us completely jobless. They were given the duty of getting the clients for the centre, now they should get them. SkyHealth provider

It was being focused again and again that you increase the number of check-ups [ANC visits] but if I could increase the number of check-ups by myself I would have increased it. But the advertisement – that was not done in the village. Advertise in the villages and make the people aware – that happened once or twice. Van came, vehicle came, but after that was happened was nothing. If a car passes by advertising – the people come down to know what is being advertised. SkyHealth provider

Qualitative findings also suggest that the branding itself was not seen as a signal of quality as women did not know the brand. In 30 interviews with women, and when talking to many more through the participant observations, not one woman mentioned how the Sky brand had attracted her to the facility. They typically reported that they had not heard of the brand. Instead, they had come to the facility on the advice of the ASHA or because they knew the health provider working there. Providers at SkyHealth centres felt that women came because of their personal reputation and not because the brand had attracted patients.

### Mechanisms for encouraging women to attend ANC services were ineffective

To establish links between SkyHealth centres and the community, SkyCare providers and ASHAs were given training and offered financial incentives to encourage women to use ANC.

Implementation of the incentives to SkyCare providers for referral to SkyHealth providers was limited (Table 4). Data from the health provider survey showed that almost no SkyCare providers reported receiving incentives for ANC referrals. In the first survey round, over half of SkyCare providers reported referring at least one woman for ANC in the past 3 months, but this fell to one third in the second round of the survey. Only 8% of SkyCare providers reported that the franchisor had set any target for ANC referrals.

**Table 4:** Provider-level measures of implementation

Indicator	Provider survey (June 2016)
A. Incentive, targets, and referrals	
SkyHealth receive incentives for ANC consultations (%)	7/23 (30%)
SkyCare receive incentives for ANC referrals (%)	1/25 (4%)
SkyHealth referred ANC clients for delivery care in past 3 months (%)	10/23 (43%)
SkyCare referred women for ANC in past 3 months (%)	8/25 (32%)
SkyHealth report having targets for ANC (%)	5/23 (22%)
SkyCare report having targets for ANC (%)	2/25 (8%)
B. Telemedicine	
SkyHealth providers ever given telemedicine consultation (%)	16/23 (70%)
Mean number of telemedicine consultations in last month	1.95 (4.7)
Telemedicine equipment works on day of interview (%)	7/23 (30%)
C. Training	
Sky providers ever trained (%)	49/49 (100%)
Duration of Sky provider training (days)	1.4 (0.6)
ASHA received training in past 12 months in intervention districts (%)	107/119 (90%)
ASHA received training in past 12 months in comparison districts (%)	55/62 (89%)
D. Supervision and monitoring	
Sky provider ever received supervision visit from WHP (%)	33/49 (67%)
Sky provider received supervision visit in the last 6 months (%)	22/49 (45%)
Mean number of supervision visits in the last 6 months (%)	1.6 (2.8)
SkyHealth received feedback on quality of care in the last 6 months (%)	12/23 (52%)

Notes: Source of data is the health provider survey (round 2, June 2016). Data are *n*/*N* (%) or mean (SD), as appropriate

The qualitative research revealed that most SkyCare providers had little or no experience working in the area of maternal health. They only interacted with pregnant women if they were sick and wanted curative care during their pregnancy. Another challenge was that SkyCare providers saw themselves as providers of care and not health educators. As a result, referring women to another facility or getting women to have a phone consultation with the central medical facility was seen as undermining their reputation as someone able to cure health problems and potentially leading to a loss of clients. As a result, some SkyCare providers saw SkyHealth providers as competition.

The women will say to you, ‘you are a doctor.’ Why are you sending me to someone else or making me talk on the phone? SkyCare provider

The relationship between the programme and ASHAs was also problematic. The franchisor initially recruited ASHAs as SkyCare providers, but this caused tension with the state government who felt that these ASHAs were a government resource. As a result, the ASHAs were quickly replaced by rural health providers, and instead, the role of ASHAs was limited to encouraging women to attend ANC and delivery care. SkyCare providers complained that working with ASHAs was challenging and that it became complicated when it came to who should get the incentives from SkyHealth providers for referring women—the ASHA or the SkyCare providers. Some SkyCare providers talked about how they had tried to organise health camps in the village with the SkyHealth provider, hoping to create linkages between the two providers. However, in order to get women to attend the health camp, they had to work with ASHAs, who would afterwards claim money for bringing women.

Finally, we found that the training of ASHAs in the past 12 months was no more common in the intervention districts than in the comparison districts. In other words, ASHAs were receiving training from a range of sources outside the programme such that Matrika was not filling a well-identified gap (Table 4).

### Referral linkages were not sufficiently strong between SkyHealth providers and Matrika delivery providers

The Matrika model relied on SkyHealth centres both to provide ANC and to refer patients to the higher-level Franchise Clinics for delivery care. One of the major challenges impeding this mechanism in practice was recruiting Franchise Clinics into the network. Indeed, 11 Franchise Clinics were expected to be referral destinations for pregnant mothers, but during data collection, it was found that only six of them were active in the Matrika programme. Moreover, the precise mechanism through which SkyHealth providers would be successful in encouraging ANC patients to give birth in Matrika delivery facilities was vague. We found from the social franchise user survey that none of the women attending SkyHealth facilities for ANC delivered in Franchise Clinics. Provider survey data showed that less than half the SkyHealth providers had referred any clients for delivery care in the past 3 months (Table 4). At one of the Franchise Clinics, the director reported not receiving any referrals from SkyHealth and SkyCare providers or ASHAs in the 2 years that he had been part of the franchise.

We wanted to get associated with someone whose network is doing well through which we could expand the hospital. I thought at this level –SHC [SkyHealth Centre] – the patient there who comes and if they have complications, they will be admitted here. But this never happened. Franchise Clinic director

Clearly, one of the major barriers to attending Franchise Clinics for delivery was the cost of care. Data from the social franchise user survey showed that the socio-economic status of users was higher at Franchise Clinics (70% in wealth quintile 5 for Uttar Pradesh as a whole and 17% in wealth quintile 4) than at SkyHealth providers where the distribution was more evenly spread across the wealth quintiles (48.5% in the top two quintiles and 16.3% in the two lowest quintiles). Given the relatively high price of delivering in Franchise Clinics (median cost of 117 USD for a normal delivery), it seemed unlikely that SkyHealth users could or would contemplate delivering there when the public sector offered free care. Additionally, the number of these facilities ended up being too low to cover a geographical area large enough for the referral mechanism to function.

### Evidence on the influence of quality improvement activities (training and supervision) on quality of care was mixed

There were a number of hypothesised pathways through which improving the capacity of health providers was anticipated to have impact. The clinical training of private providers in the social franchise network was intended to raise quality of ANC, delivery care, and post-partum family planning through increases in knowledge and skills. Quality improvement visits were also expected to increase quality of ANC and delivery care in both private and public facilities.

Various sources of data support the possibility that the clinical training at Sky facilities increased competence of ANC. Training was widely implemented. Table 4 shows that all Sky providers reported receiving training. Implementation of the supervision was, however, patchy, with only 45% of providers receiving a visit in the previous 6 months. Table 5 shows results from the health provider survey in which we measured ANC knowledge of health providers across 61 items and calculated an overall competence score, defined as the percentage of measured items answered correctly. SkyHealth providers trained under Matrika had either the same or greater ANC knowledge than their untrained counterparts. Table 5 also shows self-reported ANC practice, measured by asking health providers what they did during their last ANC consultation. We aggregated nine items to generate an overall score of ANC practice. The reported practice of SkyHealth providers was substantially better than that of equivalent providers located in the same intervention clusters or in non-intervention clusters. Multiple interpretations of these data are possible. The training or telemedicine may have improved actual practice, the private providers selected to be SkyHealth providers may have been more competent in the first place, or both.

**Table 5:** Provider antenatal care knowledge and practice

Indicator	Provider survey (June 2016)
A. Provider ANC knowledge (index score, 61 items)	
Comparison of SkyHealth and other private AYUSH providers	
SkyHealth providers	0.53 (0.11)
Private AYUSH (not SkyHealth) in intervention villages	0.35 (0.14)
Private AYUSH in comparison villages	0.23 (0.15)
Comparison of ASHAs in intervention and comparison districts	
ASHA in intervention districts	0.44 (0.10)
ASHA in comparison districts	0.43 (0.14)
B. Provider ANC practice (index score, 9 items)	
Comparison of SkyHealth and other private AYUSH providers	
SkyHealth providers	0.66 (0.31)
Private AYUSH (not SkyHealth) in intervention villages	0.26 (0.27)
Private AYUSH in comparison villages	0.15 (0.25)
Comparison of ASHAs in intervention and comparison districts	
ASHA in intervention districts	0.25 (0.25)
ASHA in comparison districts	0.28 (0.24)

Notes: Source of data is the health provider survey (round 2, June 2016). Data are mean (SD). We measured ANC knowledge of health providers across 61 questions (items). The response to each question was used to generate an overall score of knowledge, defined as the proportion of measured items answered correctly. Self-reported ANC practice was measured in the health provider survey by asking respondents to recall their last ANC consultation and what they did during the consultation. We aggregated responses to nine questions (items) to generate an overall score of ANC practice, defined as the proportion of measured items reported as practiced

However, other data suggest that monitoring and supervision visits were unlikely to have improved quality of care in Sky facilities. Qualitative responses were mixed about the adequacy of visits from WHP staff to support clinics in the SkyHealth facilities. While they acknowledged that franchisor staff visited them regularly, they said that generally these visits did not concern quality—rather WHP staff came to monitor data collection (i.e., names of maternal health patients who attended the clinic). It is possible that these visits were in fact monitoring visits and that the clinic directors simply did not perceive them to be such. In terms of routine quality of care monitoring and improvement, facility heads did not suggest that they were disappointed with the irregularity of supervision, training, feedback, and activities. In general, observations and interviews suggested that most clinic heads were satisfied with their own capacities and viewed the technical management of any clinical staff to be their own domain. For the most part, they did not show themselves to be eager for skill and knowledge improvement.

### Telemedicine was an attractive feature for clients but problems in the implementation of the technology meant its effect on quality of ANC was likely limited

Telemedicine was intended to link the patient to a qualified and experienced doctor at the central medical facility, thereby ensuring quality ANC services at SkyHealth facilities. Availability of telemedicine was also supposed to increase demand for ANC.

Various findings raise questions about the benefits of telemedicine in practice. Although telemedicine was seen as an attractive feature of what SkyHealth centres had to offer, we found important flaws in the implementation of the technology. First, multiple sources of data suggest that the telemedicine equipment did not always work. Data from the health provider survey show that only 70% of SkyHealth providers had ever used the telemedicine equipment (Table 4). Only 30% of SkyHealth providers reported that the equipment was working on the day of interview, and the average number of teleconsultations per month was 2.0 (range 0 to 20). The most common problem was internet connectivity (data not shown). The qualitative research raised similar concerns. When talking to women, ASHAs, and providers and observing telemedicine consultations, a number of problems were clear. There were often technical problems or glitches that meant the consultation did not happen or women had to wait a long time, sometimes a number of hours. Women were often a bit bewildered about “who” the doctor was and who they were talking to. In a number of clinics, there was a communication problem, as the central medical facility staff could not understand the dialect that women were speaking. Women felt it was hard to talk to someone they could just see on a screen.

The doctor [on the computer] wasn’t talking to me, she was talking to the other doctor [provider at the clinic] and that doctor was talking to me. The doctor [provider at the clinic] was asking me things and the doctor on the computer. But I couldn’t speak properly to her [the doctor on the computer] as she was from the city. So I would tell the doctor [in the clinic] and he would tell the other doctor. I could see her face but I did not pay attention as I was looking at the doctor at the facility. SkyHealth user

ASHAs also complained the telemedicine often did not work, that the consultations took too long and women became impatient.

Women are worried they had to wait at the facility for many hours. Some women would shout at me saying ‘we have spent some much time here, there is not internet, we have a lot of work at home.’ Some women would get angry at us, they would say they are losing their time because of me. Gradually I lost interest due to the refusals. ASHA

Data from clinical observations in four SkyHealth facilities suggest that the quality of ANC provided through telemedicine was highly variable (Table 6). Some key components of ANC were uniformly implemented, while many other components were rarely done. In practice, implementation of ANC through telemedicine had limitations, particularly in relation to some of the essential physical examinations. The issue was exacerbated by the fact that most SkyHealth providers were male, when women in this context prefer female health workers for ANC and maternity care services.

**Table 6:** ANC practices observed during telemedicine consultations in SkyHealth facilities

Activities performed by healthcare worker	SkyHealth
Washed hands before procedures	0/25 (0%)
Put on examination gloves	0/25 (0%)
Weighed the client	25/25 (100%)
Took the client’s blood pressure	25/25 (100%)
Examined hands for oedema	25/25 (100%)
Performed or referred for urine test	0/25 (0%)
Tested for proteinuria	0/25 (0%)
Tested for glucose	0/25 (0%)
Checked for sign of anaemia	0/25 (0%)
Performed or referred for anaemia test	2/25 (8%)
Palpated client’s abdomen for fundal height	0/25 (0%)
Listened for foetal heartbeat	2/25 (8%)
Performed or referred for syphilis test	0/25 (0%)

Notes: Source of data is the clinical observations of antenatal care consultations done by telemedicine. Data are *n*/*N* (%)

## Discussion

This process evaluation of the Sky social franchise was conducted alongside an impact evaluation, which measured a wide range of study outcomes, covering healthcare utilisation, quality of care, patient experience, healthy behaviours, health status, and financial strain [12]. The findings from the impact evaluation showed that there was no evidence that the multi-faceted project had a population impact on the vast majority of outcomes measured, with the exception of a small effect on recommended delivery care practices. Notably, the programme was shown to have no effect on ANC utilisation, ANC content of care, and facility births.

Our approach in the process evaluation was to develop a theory of change to map out the key mechanisms linking inputs and outputs and then to critically assess the logic of the proposed mechanisms, their extent of implementation, and whether they worked in practice. Using the framework provided by the theory of change, we reported six key findings that help explain why the social franchise model was not effective in improving the quality and coverage of maternal health services. First, the franchisor achieved its health provider recruitment targets but the competitive nature of the market for antenatal care meant social franchise providers achieved very low market share. Moreover, the fact that most providers were male may have made the SkyHealth centres a less attractive option. Second, despite the Sky providers being branded, community awareness of the franchise was low and the brand was not perceived as a signal of quality. Third, mechanisms for encouraging women to attend antenatal care services were ineffective. Fourth, referral linkages were not sufficiently strong between antenatal care providers in the franchise network and delivery care providers. Fifth, evidence on the influence of quality improvement activities (training and supervision) on quality of care was mixed. Finally, telemedicine was perceived by clients as an attractive feature, but problems in the implementation of the technology meant its effect on quality of antenatal care was likely limited.

The study had a number of limitations. First, the evaluation focused on the Sky social franchise component of what was a multi-faceted intervention and did not, for example, examine project activities in the public sector. Second, the process evaluation included multiple sources of data that were collected at different points in time in an evolving programme. Indeed, some components of the programme that did not work as intended were recognised by the franchisor, who made adjustments during the course of implementation. At the same time, a strength of our study was the triangulation of different data sources that enabled us to have an in-depth understanding of the design, implementation, and context of the programme. Moreover, the quantitative and qualitative research teams coordinated closely to ensure that the two types of data built on each other and avoided duplication. Third, the analysis and interpretation of data from the process evaluation took place after preliminary findings from the impact evaluation were known. It has been noted that an approach in which the two types of evaluation are conducted independently, blinded from each other’s findings, may be less prone to bias [16]. Finally, some of our quality of care measures were based on self-reports with potential recall problems, and while direct observations are preferable from a methodological standpoint, we conducted only a small number.

The findings pertain to this particular social franchising model, and we note that other social franchising programmes for maternal health are designed quite differently. For example, the Merrygold social franchise in neighbouring Rajasthan seeks to engage only with higher volume delivery care providers in the private sector. On the other hand, there were mechanisms within Matrika that are common to other social franchise models, such as the training, quality improvement support, branding and marketing, and demand generation using community health workers, meaning that our findings raise a number of questions for most clinical social franchising programmes. Although our findings on quality of care were inconclusive, the training of two to three days was shorter than what evidence suggests is necessary to improve practice [21–23]. There is the question of whether patients recognise the brand of the social franchise as a signal of quality, particularly in contexts where the reputation of the provider is paramount. Other social franchise studies similarly reported that branding and marketing activities had little impact on influencing care-seeking behaviour [24, 25]. Relying on government community health workers to generate demand for social franchise services may be optimistic, as documented elsewhere [26, 27]. Also, to assess whether telemedicine can improve quality of care, one must consider the appropriateness of the technology with respect to specific health services and the available infrastructure. Finally, we note that firms in a commercial franchise model are incentivised to maintain quality standards through the threat of expulsion from the franchise and the subsequent loss of business. This mechanism was not a key feature of the Sky franchise model, in its design or implementation. It is debatable whether social franchisors can in practice leverage such incentives, especially when they are looking to meet recruitment targets and, if not, what the implications are for their success.

The Sky franchise model for maternal health in Uttar Pradesh was adapted from an earlier programme focused on child health in Bihar. At the time, the model was seen as quite successful based on programme implementation data, though a rigorous evaluation later found no impact [28]. Moreover, our results highlight some of the pitfalls of transporting mechanisms from one disease area to another without appropriate adaptation. Maternal health services, and particularly delivery care, require a more sophisticated level of skill and equipment than most ambulatory care for childhood diseases, meaning that improving quality was much more challenging and that village-level providers were ultimately not deemed appropriate as delivery providers. In addition, the challenge of maintaining continuity of care during pregnancy and identifying women at high risk was perhaps under-appreciated.

The broader markets for maternal care and curative care are also quite different, highlighting the importance of contextual factors in explaining intervention impact. An underlying assumption of the Sky model, perhaps transferred from the Bihar model, was that as rural health providers are often the only health providers in the vicinity of a village and are frequently used, it made sense to work with these providers to train them to perform ANC and encourage them to refer women for delivery. However, while these rural health providers were also widely used in Uttar Pradesh, they were not the usual choice for maternal healthcare or for health promotion activities, and it was difficult to shift these care-seeking patterns. In addition, the competitive nature of the market explained the low market share achieved by social franchise providers, a finding that is consistent with an evaluation of the franchising model in Bihar [28]. Public sector providers had a distinct competitive advantage through a number of existing policies, including (1) the Janani Suraksha Yojana (JSY) incentive scheme, which provided financial incentives to ASHAs and women when the women sought care in public hospitals for ANC and delivery [29]; (2) free ambulance services for transport to public facilities [30]; and (3) free public ANC care. Other influential contextual factors included difficulties in recruiting female health providers into the network and the existing infrastructure that meant the telemedicine technology did not function as well as intended.

The evaluation has clear lessons for project design. Specifically, at the design stage of a project, it can be extremely useful for implementers to develop a theory of change and critically probe each of the intended pathways of impacts. This process can shed light on the logic and coherence of the programme. There were a number of components of the Sky social franchise that were poorly conceived. In some cases, it is with the benefit of hindsight that we can say this—e.g., the market for ANC was more competitive than first assumed. But in other cases, components were not sufficiently thought through at the outset—e.g., women using SkyHealth providers were unlikely to be able to afford the prices charged for delivery care at Franchise Clinics. Probing the intended pathways of impact would also require formative research and small-scale piloting prior to implementation in order to understand market conditions, what patients value, and how new technologies perform.

Although caution should always be exercised when generalising findings, our focus on mechanisms in process evaluation should give the study greater external validity. At the very least, the findings should place a higher burden of proof on policymakers and funders who propose investing in social franchising for maternal health. Relative to other studies on social franchising, the evaluation of this social franchise is at the rigorous end of the spectrum; the existing evidence on social franchising in LMICs remains weak [7], and engagement with the private sector through social franchising appears to be moving in advance of evidence. However, there is also a lack of evidence on alternative effective and cost-effective strategies to improve quality amongst these providers, indicating that further innovation and experimentation should be encouraged. As institutional delivery rates continue to rise, the question of how to improve quality should be at the forefront of policymakers’ minds. This is an area of research that needs much more attention, combining the expertise of clinicians, social scientists, management scientists, and other disciplines.

## Conclusion

The findings presented in this process evaluation offer key insights into *why* the social franchise model did not improve the quality and coverage of maternal health services. They point towards the importance of designing programmes based on a strong theory of change, understanding market conditions and what patients value, and rigorously testing new technologies. The design of future social franchising programmes should take account of the challenges documented in this and other evaluations.

## Footnotes

1 Franchise Clinics consisted of privately run facilities as well as two facilities owned and administered by WHP
